# Myopia information on TikTok: analysis factors that impact video quality and audience engagement

**DOI:** 10.1186/s12889-024-18687-4

**Published:** 2024-04-29

**Authors:** Shuai Ming, Jie Han, Xi Yao, Xiaohong Guo, Qingge Guo, Bo Lei

**Affiliations:** 1 Henan Eye Institute, Henan Eye Hospital, Henan Provincial People’s Hospital, Zhengzhou, Henan 450003 China; 2 Eye Institute, Henan Academy of Innovations in Medical Science, Zhengzhou, Henan 451163 China; 3 Henan Clinical Research Center for Ocular Diseases, People’s Hospital of Zhengzhou University, Zhengzhou, Henan 450003 China; 4 School of Business, Zhengzhou University of Aeronautics, Zhengzhou, Henan 450015 China

**Keywords:** TikTok, Myopia, Information quality, Public education, Social media, Audience engagement

## Abstract

**Background:**

TikTok is emerging as a vital platform for health information dissemination. Despite myopia being a global public health issue, the high-quality myopia information shared by health educators often fails to go viral. It is imperative to analyze the factors influencing video quality and popularity, especially from diverse perspectives of researchers, health educators, and audiences.

**Methods:**

TikTok myopia-related videos were retrieved using TikTok’s default comprehensive search (DCS) and most liked search (MLS) strategies. Venn diagrams were employed to illustrate the relationships and commonalities between the two strategies across four sample sizes (top 200, 150, 100, and 50). Video metadata, including details such as creator information, production properties, upload time, video duration, and viewer engagement, were collected. Video quality was assessed using the DISCERN tool. Video content covering six aspects of myopia were evaluated. The impact of search strategies, video sample sizes, production properties, and myopia content on video quality and audience engagement was analyzed through single-factor or multi-factor analysis.

**Results:**

DCS and MLS retrieval strategies, as well as varying sample sizes, resulted in differences in audience engagement for myopia videos (*P* < 0.039), while The DISCERN quality scores remained comparable (*P* > 0.221). Videos published by healthcare professionals (HCPs) and non-profit organizations (NPOs) were associated with high-quality (*P* ≤ 0.014) but comparatively lower popularity (*P* < 0.033). Videos that reported contents of risk factors, management, and outcomes showed high popularity (*P* < 0.018), while longer video duration (> 60s) exhibited the opposite trend (*P* < 0.032). Content on myopia evaluation (*P* ≤ 0.001) and management (*P* ≤ 0.022) and video duration were positively correlated with higher DISCERN quality.

**Conclusion:**

Videos created by HCPs and NPOs deserve greater attention. Rather than pursuing entertaining effects, professional educators should emphasize producing concise, and high-quality myopia content that readily resonates with the audience and has the potential to go viral on the platform.

**Supplementary Information:**

The online version contains supplementary material available at 10.1186/s12889-024-18687-4.

## Introduction

Myopia is emerging as a global visual health issue affecting the daily lives of billions worldwide [1, 2]. Individuals with myopia commonly experience challenges in long-distance vision. Moreover, myopia substantially contributes to uncorrected refractive error, a leading cause of visual impairment [3]. Complications associated with myopia, particularly high myopia, such as glaucoma, retinopathy, and retinal detachment, are also significant causes of blindness [4]. It was estimated that the global myopia prevalence might increase from 1.4 billion in 2000 to 4.8 billion by 2050 [5]. Without scientific management and interventions, this issue could lead to a substantial global disease burden [6, 7]. Therefore, educating the public about myopia and raising awareness of preventive measures is particularly important.

Social media has become an important channel for disseminating medical health information [8]. TikTok, known as a globally popular short-video social media platforms, has over 1.1 billion monthly active users globally [9]. Previous reports suggested that TikTok might serve as an effective platform for health education in the digital age, given its unique format of short, engaging videos and its large user base [10]. The research agenda regarding TikTok has also been raised by healthcare experts [11]. In fact, medical professionals are the driving force for creation of educational myopia videos creation on TikTok [12]. Numerous ophthalmologists, optometrists, and institutions have utilized the platform to release educational video content about myopia prevention and treatment, aiming to broaden its reach and enhance public health awareness. However, videos produced by medical professionals often exhibit a notable deficiency in terms of popularity and audience engagement [13]. Therefore, it is urgent for eye care providers to understand what types of myopia videos are more apt for dissemination and possess higher audience engagement. Research on engagement specifically related to myopia videos is lacking.

Audiences frequently search for health-related information on the TikTok platform, and this especially was true during the COVID-19 pandemic [14, 15]. Our previous investigation indicated that the quality of myopia-related videos on TikTok was generally subpar, with noticeable disparities in video quality across various sources [12]. Similar findings were also reported regarding TikTok information related to other diseases [16–18]. Some disease videos even exhibit a notable prevalence of misinformation, ranging from 41.2% to 77.8% [19–21]. These findings present challenges for viewers in discerning and selecting high-quality myopia education videos. While a previous study explored the quality of Mpox information on TikTok based on video characteristics and content [22], there is limited research on myopia information. Furthermore, external factors such as TikTok’s default retrieval algorithm, audience personal preference for videos with more likes, and the sample size for quality evaluation might also influence the information encountered by viewers. However, no study has investigated whether such choices result in differences in the quality of myopia videos presented to viewers.

This study investigated the potential impact of search strategies and diverse samples on video quality and audience engagement. Furthermore, we explored potential contributing factors related to video production characteristics and myopia content. We aim to address researchers’ concerns regarding selection bias in video selection, and further provide insights for healthcare providers to create appealing content and guide viewers in discovering high-quality myopia educational videos.

## Methods

### Search strategies

This was a cross-sectional study. The search was conducted on March 12, 2022. Given TikTok’s user-driven nature, the free-word search method was favored over the “#topic label” approach. The term “myopia” was input into search box (TikTok Chinese version 20.2.0), generating a list based on the TikTok Default Comprehensive Search (DCS) strategy. This adaptive algorithm, tailored to user preferences, aimed to present videos deemed most suitable for viewers [10]. The DCS was believed to be the primary method viewers used for video retrieval. TikTok also provides an option to sort by “most liked”. The “most liked” search (MLS) strategy, based on total like counts, is believed to guide viewers to popular videos. Metadata from videos retrieved via DCS and MLS were collected and documented in MS Excel, capturing details like creator information, upload time, video duration, and viewer engagement.

**Fig. 1:**
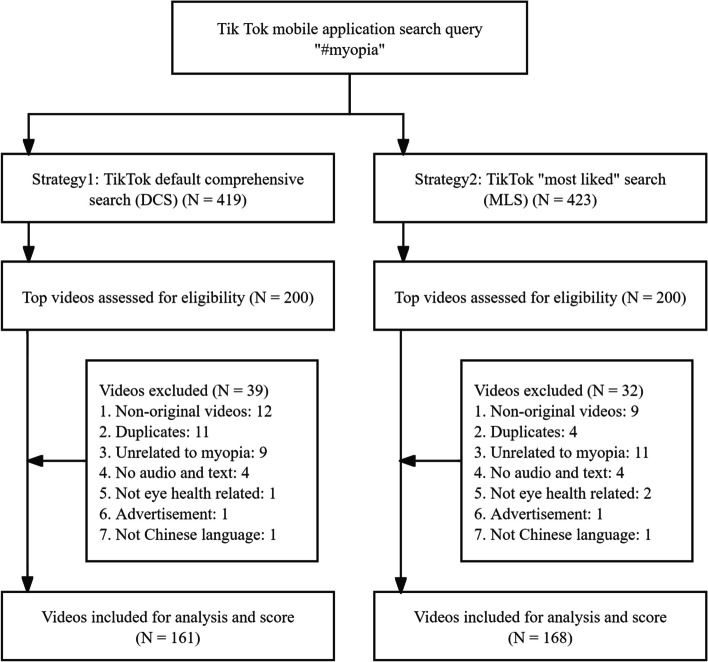
Flow chart diagram of the top-200 videos retrieved from DCS and MLS

**Fig. 2:**
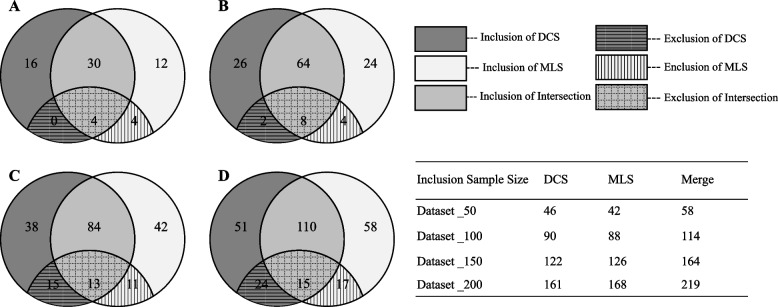
Venn diagrams and tables of inclusion numbers for two strategies (DCS and MLS). **A** Venn diagram of sample size 50. **B** Venn diagram of sample size 100. **C** Venn diagram of sample size 150. **D** Venn diagram of sample size 200. DCS: default comprehensive search strategy. MLS: “most liked” search strategy

The TikTok users spend an average of 95 min daily on the platform [9]. Assuming a 30-s duration for each short video, it is not likely that users would browse more than 200 videos during their daily fragmented periods. Therefore, for both strategies, the initial selection for further screening included the top 200 myopia-related videos. After excluding material considered to be non-original, duplicate, irrelevant to myopia, and videos without audio narration or text, a total of 161 and 168 videos were finalized for the DCS and MLS strategy groups, referred to as Data_DCS_200 and Data_MLS_200, respectively (see search strategies in Fig. 1). Additionally, we organized the inclusion of videos for each strategy’s top 150, 100, and 50 records, as well as Venn diagrams for the four sample sizes of both strategies (Fig. 2).

## Quality assessment

Video quality was assessed using the DISCERN tool [23], which includes 16 items evaluating “reliability” (items 1–8), “treatment choices” information quality (items 9–15), and “overall quality” (item 16). Items 1—15 are coded using a 5-point Likert scale where 1 = “No” (not meeting the criterion), 3 = “Partially” (partly meeting the criterion), and 5 = “Yes” (fully meeting the criterion). Item 16 utilizes a 5-point Likert scale where 1 = “Low, serious shortcomings”, 3 = “Moderate, potentially important but not serious shortcomings”, and 5 = “High, minimal shortcomings”. A higher score indicated high quality within each domain. Not all videos contained treatment information; however, according to the DISCERN tool manual, content related to personal care measures for myopia prevention, such as increasing outdoor activities and reducing near work time, should be considered therapeutic intervention and be included in the treatment choice domain. Total DISCERN quality was classified as very poor (< 26), poor (27–38), fair (39–50), good (51–62), and excellent (63–80) [17, 22, 24].

Assessments were independently conducted by two experts, an ophthalmologist and an eye public health physician (XK and MS), who utilized the guidelines provided in the official DISCERN handbooks. Both assessors achieved an initial agreement on the interpretation of these guidelines. They were restricted to viewing only the videos without exposure to any information about the video authors or their classifications, to prevent any selection bias. Any divergences in their evaluations were discussed and settled amicably.

## Video popularity and audience engagement

The metadata of greater video likes, comments, shares, and saves received as of the retrieval date were indicative of greater video popularity and audience engagement. Additionally, the ratio of video likes to the number of days since publication could serve as an incremental indicator of the rate of likes and reflect the overall video engagement. Subsequently, video likes, shares, and the ratio of likes to days were employed as the independent variables for factor analysis, as they exhibited a normal distribution after log transformation.

## Video features and content

The primary video features included the presence of people, medical science education labeling, background music, emojis, animation flashes, video duration, and video source [22]. The video source specified the type of publisher, including healthcare professionals (HCPs), individual science communicators (ISCs), for-profit organizations (FPOs), non-profit organizations (NPOs), and news agencies (NAs) [25]. We combined NAs into the NPOs category. Detailed definitions can be found in our previous study [12].

The video content encompassed six aspects: definition, symptoms, risk factors, assessment, management, and outcomes of myopia. The specific content can be found in our previous research [12] and combined with the International Myopia Institute (IMI) series of articles updated in 2023 [26–28]. Each aspect was scored as follows: 0 for “Not mentioned”, 1 for “Partially mentioned”, and 2 for “Fully mentioned”. An aspect was considered “Fully mentioned” if the video covered more than three topics within that aspect; otherwise, it was rated as “Partially mentioned” or “Not mentioned”.

## Data analysis

**Fig. 3:**
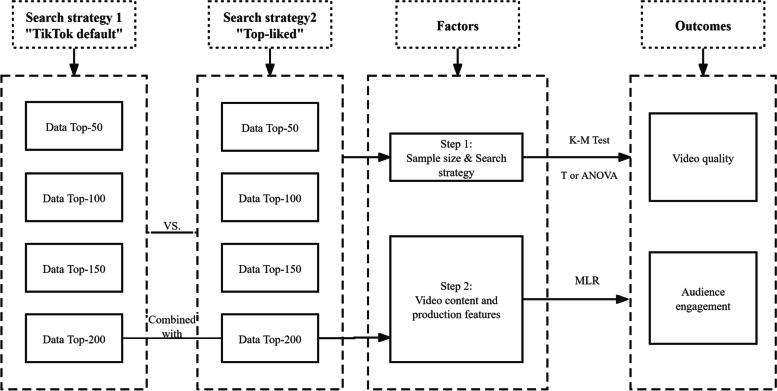
The design and analytical framework of the study

For statistical description, variables related to video quality were presented as mean ± standard deviation, while audience engagement related variables were expressed as median (P_25_, P_75_). Video content and features were denoted as n (%). Statistical analysis was divided into two steps. Step 1: We analyzed the impact of search strategies and video sample sizes on video quality and audience engagement. T-tests or Kruskal–Wallis tests were used to compare videos retrieved through DCS and MLS searches. ANOVA or Kruskal–Wallis tests were applied to assess differences in quality and popularity among the top 50, 100, 150, and 200 videos, adjusting for inclusion criteria. For multiple comparisons, the Bonferroni correction method was applied to adjust significance levels. Step 2: The combined set of the Data_DCS_200 and Data_MLS_200 was employed to explore the influence of video content and production features on video quality and audience engagement using multiple linear regression (MLR). Variables underwent log-transformation to fulfill the normal distribution assumption of the dependent variable, where Log(video likes), Log(shares), and Log(video likes/publish days) were used as indicators for audience engagement. The six content areas were treated as ordinal variables. Video sources and video duration were dummy-coded, with “ISCs & FPOs” and “ < 30 s” serving as reference categories respectively. Given the inclusion of dummy variables, requiring simultaneous entry and removal in the model, the variable selection method chosen for MLR analysis was “Enter”. The level of statistical significance was set at 0.05. The specific approach and analytical framework are depicted in Fig. 3.

## Results

### Video characteristics and contents

The combined data-set of Data_DCS_200 and Data_MLS_200 encompassed 219 videos, of which 130 videos reported information about treatment options. Nearly half (47.9%, 105/219) of the videos were published by health professionals. The median duration of the videos was 62 s. Most videos (69.4%, 152/219) featured real people delivering the information, while only 30.6% (67/219) included education labels and approximately 29.7% (65/219) and 48.9% (107/219) had emojis and animations, respectively (Table 1).

**Table 1:** Video production properties

Video production properties	Videos (*N* = 219)	Videos with treatment choices (*N*' = 130)
With people presence	152 (69.4)	97 (74.6)
Marking with education labels	67 (30.6)	40 (30.8)
With background music	138 (63.0)	80 (61.5)
With emoji effects	65 (29.7)	60 (46.2)
With animation flash	107 (48.9)	41 (31.5)
Video publisher		
ISCs & FPOs	65 (29.7)	36 (27.7)
NPOs	49 (22.4)	33 (25.4)
HCPs	105 (47.9)	61 (46.9)
Video duration	62 (35.5, 9.5)^a^	70 (46, 99)^a^
≥ 120 s	32 (14.6)	26 (20)
60—119 s	80 (36.5)	54 (41.5)
30—59 s	76 (34.7)	41 (31.5)
< 30 s	31 (14.2)	9 (6.9)

For the categorical variables of “Video publisher” and “Video duration”, the proportions represented in n (%) were column percentages

*ISCs* Individual science communicators, *FPOs* For-profit organizations, *NPOs* Non-profit organizations, *HCPs* Healthcare professionals, Data (*N*' = 130) was used for analyzing the influencing factors of both the quality dependent variables of “Treatment” and “Total”

^a^data were showed as Median (P_25_, P_75_)

**Fig. 4:**
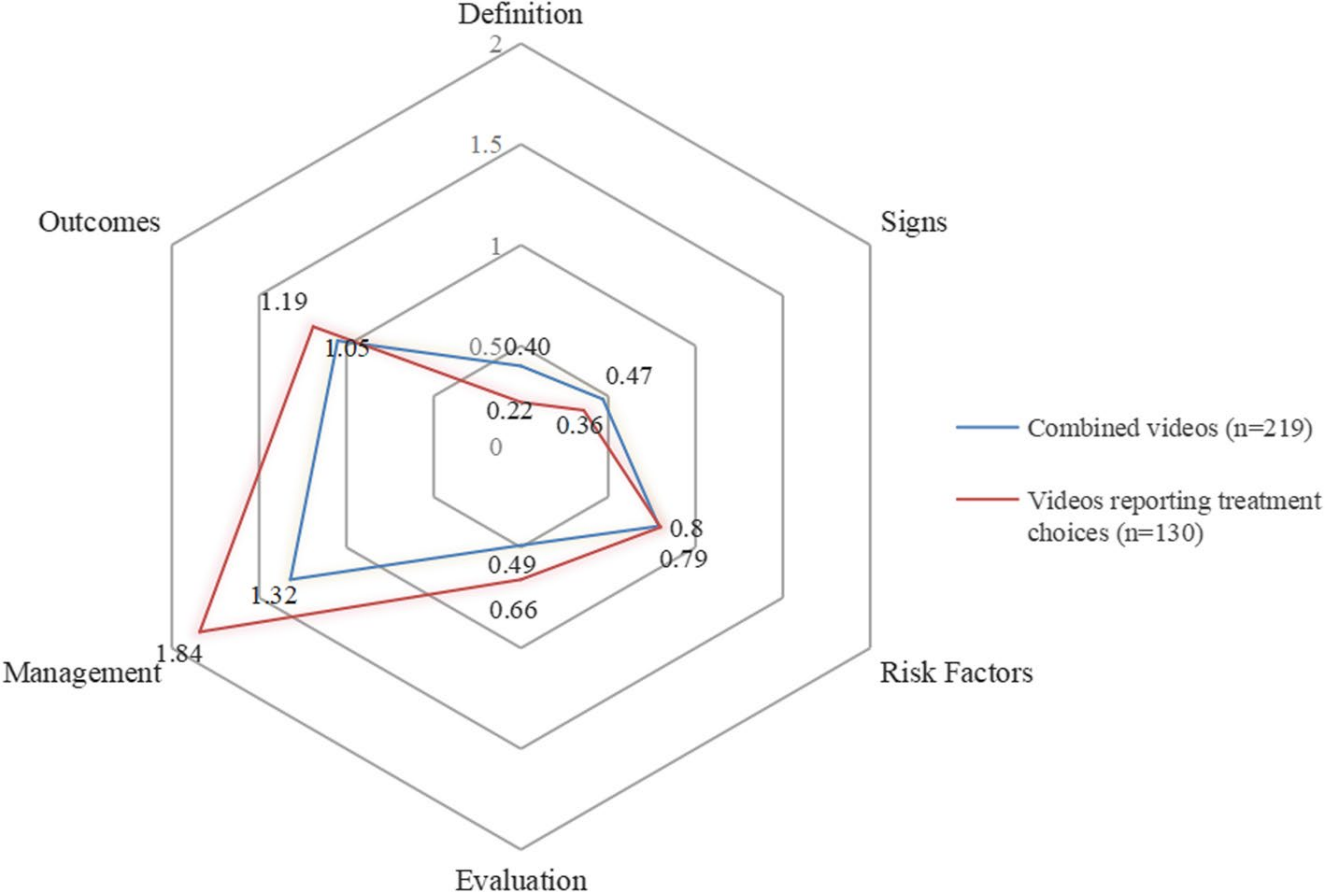
The mean score of each myopia content domain

In terms of video content, the domains of myopia management (76.3%) and outcomes (7.8%) were more frequently reported or partially reported by the video publishers. Conversely, nearly 70% of the videos neglected information about the definition and signs of myopia. The mean score of each content domain is shown in Fig. 4.

### Audience engagement and video quality

The combined dataset of 219 videos, over a period of 223 (143.5, 296.5) days, accumulated likes of 2.25 million. The median likes and shares were 819 (253.5, 3566.5) and 146 (38.0, 652.5) respectively. In the DISCERN quality assessment, the reliability domain scored (19.73 ± 4.13) with a 95% CI of 19.18 to 20.28, the Treatment choice domain scored (17.28 ± 4.04) with a 95% CI of 16.58 to 17.99. Additionally, the total score was (41.30 ± 8.38) with a 95% CI of 39.85 to 42.75. The Total quality was fair. No significant association was found between audience engagement and video quality (Table 2).

**Table 2:** Association analysis between DISCERN domains and audience engagement

DISCERN domain		**Log (likes)**	Log **(likes/days)**	**Log (shares)**
Reliability	r (P)	0.070 (0.300)	0.054 (0.425)	0.116 (0.093)
Treatment	r (P)	-0.005 (0.951)	0.006 (0.947)	-0.066 (0.464)
Total	r (P)	0.036 (0.681)	0.044 (0.620)	-0.025 (0.785)

There were no statistically significant differences in the DISCERN quality scores (reliability, treatment, and total score) for myopia-related videos obtained through the two search strategies (DCS and MLS) and across the data of four sample sizes (50/100/150/200) (*P* > 0.221) (see Table 3). Apparently, the DCS search strategy showed lower audience engagement compared to MLS (*P* < 0.039). Although not all pairwise comparisons revealed differences, an overall decreasing trend in audience engagement was observed with the DCS strategy as sample sizes increased (*P* < 0.001) (see in Supplemental Table S1).

**Table 3:** Audience engagement and video quality from two search strategies and different sample size

Outcomes	Sample_50	Sample_100	Sample_150	Sample_200	*P*
N of Likes—DCS	6828 (910, 17,190)^a^	3614 (910, 2498)^a^	2269 (321, 5527)^a^	864 (144, 3961)^a^	< 0.001
N of Likes—MLS	17,450 (9229, 51,361)	4545 (2498, 16,912)	2731 (1113, 9229)	1554 (531, 5073)	< 0.001
N of Comments—DCS	102 (31, 610)^a^	102 (23, 314)^a^	68 (9, 236)^a^	28 (5, 147)^a^	< 0.001
N of Comments—MLS	595 (183, 1676)	201 (71, 611)	111 (46, 301)	69 (21, 227)	< 0.001
N of Shares—DCS	825 (167, 3416)^a^	612 (126, 1305)^a^	222 (59, 953)^a^	120 (23, 739)^a^	< 0.001
N of Shares—MLS	3392 (797, 6365)	829 (226, 2705)	449 (146, 1305)	243 (91, 886)	< 0.001
N of Saves—DCS	237 (82, 799)^a^	139 (32, 345)^a^	88 (11, 240)^a^	28 (5, 157)^a^	< 0.001
N of Saves—MLS	858 (281, 2381)	224 (87, 799)	132(41, 345)	73 (27, 237)	< 0.001
DISCERN Reliability—DCS	19.43 ± 4.04	19.42 ± 3.78	19.41 ± 3.91	19.40 ± 3.92	0.998
DISCERN Reliability—MLS	20.29 ± 4.70	20.05 ± 4.77	19.83 ± 4.53	19.79 ± 4.37	0.902
DISCERN Treatment—DCS	16.83 ± 4.70	16.90 ± 4.38	16.92 ± 4.18	16.87 ± 4.16	0.999
DISCERN Treatment—MLS	17.09 ± 4.76	17.05 ± 4.38	17.20 ± 4.17	17.23 ± 4.05	0.993
DISCERN Quality—DCS	2.97 ± 1.00	2.97 ± 0.96	3.00 ± 0.94	2.96 ± 0.92	0.993
DISCERN Quality—MLS	3.00 ± 1.02	3.05 ± 1.00	3.10 ± 0.95	3.12 ± 0.93	0.916
DISCERN Total—DCS	40.03 ± 8.59	40.05 ± 8.21	40.32 ± 8.25	40.08 ± 8.32	0.997
DISCERN Total—MLS	41.06 ± 9.30	41.02 ± 9.14	41.22 ± 8.62	41.30 ± 8.48	0.999

*DCS* Default Comprehensive Search, *MLS* Most Liked Search

^a^represented the comparisons of each outcome between the two search strategies; *P* represented the parameter comparisons across the four sample sizes

### Factors associated with audience engagement

Videos featuring the presence of people tended to receive more likes (*P* = 0.003) and gain likes more quickly (*P* = 0.001). Conversely, videos published by HCPs and NPOs were associated with lower levels of both Log(likes) and Log(video likes/days) (*P* < 0.033). Video duration exceeding 60 s, particularly those ≥ 120 s, were associated with lower level of Log(video likes/days) (*P* < 0.032). Video content that reported information on domains of risk factors, management, and outcomes was more likely to be liked and shared (*P* < 0.018) (See Table 4). The summary statistics (β) of the untransformed data for each audience engagement variable was showed in Supplemental Table S2.

**Table 4:** Factors associated with audience engagement fixed by Multiple Linear Regression (MLR) model

Variables	Log (likes)		Log (likes/days)		Log (shares)	
	β (95% CI)	*P*	β (95% CI)	*P*	β (95% CI)	*P*
Video property (Yes = 1, No = 0)						
People presence	1.331 (0.473 to 2.190)	0.003	1.389 (0.555 to 2.222)	0.001	0.598 (-0.245 to 1.441)	0.163
Marked education	-0.198 (-0.846 to 0.450)	0.547	-0.392 (-1.021 to 0.237)	0.221	-0.394 (-1.029 to 0.242)	0.224
Background Music	-0.135 (-0.797 to 0.527)	0.688	-0.240 (-0.882 to 0.402)	0.463	0.094 (-0.556 to 0.744)	0.776
Emoji	0.274 (-0.431 to 0.980)	0.444	0.340 (-0.345 to 1.025)	0.328	-0.178 (-0.865 to 0.510)	0.610
Animation/flash	-0.249 (-1.011 to 0.513)	0.52	-0.265 (-1.005 to 0.475)	0.481	-0.592 (-1.336 to 0.152)	0.118
Video source						
ISCs & FPOs (Ref.)	0	NA	0	NA	0	NA
HCPs	-1.857 (-2.667 to -1.047)	< 0.001	-1.425 (-2.211 to -0.639)	< 0.001	-1.585 (-2.378 to -0.791)	< 0.001
NPOs	-0.838 (-1.639 to -0.036)	0.041	-0.903 (-1.680 to -0.125)	0.023	-0.429 (-1.221 to 0.363)	0.286
Video duration						
< 30 s (Ref.)	0	NA	0	NA	0	NA
30—59 s	0.029 (-0.869 to 0.928)	0.949	-0.425 (-1.298 to 0.447)	0.338	-0.188 (-1.064 to 0.689)	0.673
60—119 s	-0.567 (-1.483 to 0.349)	0.223	-0.974 (-1.863 to -0.085)	0.032	-0.369 (-1.265 to 0.527)	0.418
≥ 120 s	-1.768 (-2.938 to -0.597)	0.003	-1.797 (-2.933 to -0.661)	0.002	-1.096 (-2.264 to 0.072)	0.066
Video content (2 = Fully mentioned, 1 = Partially mentioned, and 0 = Not mentioned)						
Definition	0.218 (-0.267 to 0.702)	0.377	-0.023 (-0.494 to 0.447)	0.922	0.018 (-0.456 to 0.493)	0.939
Signs	0.434 (0 to 0.868)	0.050	0.339 (-0.082 to 0.76)	0.114	0.145 (-0.277 to 0.567)	0.498
Risk Factors	0.474 (0.126 to 0.822)	0.008	0.306 (-0.032 to 0.644)	0.075	0.398 (0.058 to 0.738)	0.022
Evaluation	0.020 (-0.374 to 0.415)	0.919	0.155 (-0.228 to 0.538)	0.426	-0.152 (-0.539 to 0.235)	0.441
Management	0.679 (0.269 to 1.089)	0.001	0.383 (-0.015 to 0.782)	0.059	0.496 (0.087 to 0.905)	0.018
Outcomes	0.764 (0.350 to 1.178)	< 0.001	0.816 (0.414 to 1.219)	< 0.001	0.774 (0.368 to 1.181)	< 0.001

*HCPs* Healthcare professionals, *NPOs* Non-profit organizations, *ISCs & FPOs* Individual science communicators and for-profit organizations, *Ref* Reference, *NA* Not available

### Factors associated with DISCERN quality

Video characteristics and content types showed varying associations with DISCERN quality. Videos from HCPs scored higher in all DISCERN domains than those from ISCs & FPOs (*P* ≤ 0.004), as did videos from NAs & NPOs (*P* ≤ 0.014). Longer videos, especially those over 60 s (*P* ≤ 0.033) and 120 s (*P* < 0.001), were associated with higher scores. Content on evaluation (*P* ≤ 0.001) and management (*P* ≤ 0.022) also correlated with higher DISCERN quality across all domains (See Table 5).

**Table 5:** Factors associated with video DISCERN quality fixed by multiple linear regression model

Variables	Reliability domain		Treatment domain		Total domain	
	β (95% CI)	*P*	β (95% CI)	P	β (95% CI)	*P*
Video property (1 = Yes, 0 = No)						
People presence	0.034 (-1.266 to 1.334)	0.959	-0.590 (-2.318 to 1.138)	0.500	-1.114 (-4.553 to 2.325)	0.522
Marked education	0.365 (-0.615 to 1.346)	0.463	0.129 (-1.099 to 1.357)	0.835	0.194 (-2.249 to 2.637)	0.875
Background Music	0.043 (-0.959 to 1.045)	0.932	0.451 (-0.817 to 1.718)	0.482	0.561 (-1.961 to 3.083)	0.660
Emoji	-0.259 (-1.327 to 0.809)	0.633	-0.095 (-1.529 to 1.338)	0.895	-0.474 (-3.326 to 2.378)	0.743
Animation/flash	0.625 (-0.529 to 1.779)	0.287	0.181 (-1.299 to 1.662)	0.809	0.48 (-2.465 to 3.426)	0.747
Video source						
ISCs & FPOs (Ref.)	0	NA	0	NA	0	NA
HCPs	2.025 (0.799 to 3.251)	0.001	2.825 (1.201 to 4.449)	0.001	5.023 (1.791 to 8.256)	0.003
NPOs	3.523 (2.311 to 4.736)	< 0.001	2.006 (0.408 to 3.604)	0.014	4.721 (1.541 to 7.901)	0.004
Video duration						
< 30 s (Ref.)	0	NA	0	NA	0	NA
30—59 s	0.140 (-1.221 to 1.501)	0.840	1.935 (-0.267 to 4.137)	0.084	3.596 (-0.786 to 7.978)	0.107
60—119 s	1.507 (0.120 to 2.894)	0.033	3.748 (1.562 to 5.935)	0.001	6.801 (2.45 to 11.153)	0.002
≥ 120 s	3.353 (1.581 to 5.125)	< 0.001	6.241 (3.751 to 8.731)	< 0.001	10.46 (5.952 to 14.967)	< 0.001
Video content (2 = Fully mentioned, 1 = Partially mentioned, and 0 = Not mentioned)						
Definition	0.068 (-0.665 to 0.802)	0.854	0.509 (-0.606 to 1.624)	0.368	0.078 (-2.141 to 2.297)	0.945
Signs	-0.150 (-0.807 to 0.507)	0.653	-0.221 (-1.154 to 0.712)	0.640	0.576 (-1.281 to 2.433)	0.540
Risk Factors	0.426 (-0.100 to 0.953)	0.112	-0.359 (-1.003 to 0.285)	0.272	-0.064 (-1.346 to 1.217)	0.921
Evaluation	1.552 (0.954 to 2.149)	< 0.001	1.260 (0.541 to 1.980)	0.001	3.140 (1.708 to 4.572)	< 0.001
Management	1.165 (0.544 to 1.786)	< 0.001	1.832 (0.265 to 3.399)	0.022	5.373 (2.255 to 8.491)	0.001
Outcomes	0.322 (-0.306 to 0.949)	0.313	0.997 (0.213 to 1.781)	0.013	1.492 (-0.069 to 3.052)	0.061

*HCPs* Healthcare professionals, *NPOs* Non-profit organizations, *ISCs* & *FPOs* Individual science communicators and for-profit organizations, *Ref* Reference, *NA* Not available

## Discussion

The public benefits significantly from the information presented in high-quality and engaging TikTok videos related to myopia. In our study, we explored the impact of search strategy and sample size on video quality and audience engagement. Interestingly, we found that these two factors influenced audience engagement but did not affect video quality. Furthermore, specific characteristics of the videos emerged as potential contributors to both video quality and audience engagement. Our research holds significance in guiding viewers to discern high-quality, evidence-based myopia content and assisting healthcare professionals, especially ophthalmologists and optometrists, in optimizing their videos for maximum impact and reach.

### Video quality and audience engagement

TikTok videos related to diabetes and chronic obstructive pulmonary disease was considered acceptable (mean total score: 47.1) or satisfactory (mean total score: 59.7) [25, 29]. In our study, the total DISCERN score of 41.3 (95% CI of 39.9 to 42.8) in our study indicated that the quality of myopia-related videos was only fair. This unsatisfactory outcome emphasized the urgent need for improvement in myopia information on TikTok. The fact that 219 myopia-related videos received 2.25 million likes and 0.2 million shares, confirmed the powerful dissemination capability of TikTok. While the Chinese version of the TikTok app cannot extract view counts, the actual audience exposed to educational information on TikTok might be largely underestimated. Therefore, TikTok emerges as a promising platform for myopia education, provided that video quality is further enhanced. This conclusion aligned with findings commonly observed in other health video research [30–32].

### Search strategy and evaluating sample size

The video retrieval process in existing literature involved both the user-preference-based MLS strategy [19, 30, 31, 33–35] and the mainly used DCS strategy [13, 16, 36–40], and four cut-off sample sizes (*n* = 50 [13, 33], 100 [16, 19, 34, 35, 38–40], 150 [30] and 200 [31, 36, 37]) were all reported. This is the first study to analyze whether these two retrieval algorithms influence the audience-available video quality and audience engagement. The MLS strategy exhibited higher audience engagement than the DCS strategy. However, for the videos retrieved by the DCS strategy, the median likes decreased from 6828 (cut-off *n* = 50) to 1554 (cut-off *n* = 200). This indicated that TikTok by default prioritized videos with higher popularity to the audience. Sun et al. [38] suggested that videos beyond the top 100 did not significantly affect the quality analysis. Our findings further extended this conclusion to the top 50. Despite differences existing in video engagement, video quality was not influenced by the MLS or DCS retrieval processes across all four included samples. This indirectly suggested the fact that varied video engagement does not correlate with video quality as reported by previous studies [18, 39]. Furthermore, these results eliminated selection bias that might be involved in the factor analysis of video quality, and further enhanced the results’ generalization.

### Video production and popularity

Video production properties were theoretically considered to potentially impact the popularity of videos [12, 41]. Our study specifically found videos featuring people were correlated with more likes and larger rate of like growth, consistent with the results of Shi et al. [22]. It indicated that users tend to prefer the direct involvement of video creators in the content dissemination process. Other production properties, as observed by Rein et al. [42], such as inclusion of education labels, background music, emojis, animation/flash did not have any correlation with the audience engagement and quality discernment of short videos. It appears that emphasizing these techniques may not necessarily enhance the popularity and quality of videos.

### Video publisher and video quality and popularity

In comparison to ISCs & FPOs, videos uploaded by HCPs exhibited higher quality but paradoxical lower levels of video audience engagement. Similar results were observed in videos uploaded by NPOs. This “quality-quantity disparity” phenomenon had been reported in other studies as well [12, 13]. The overall quality of myopia-related videos on TikTok was already inherently low. Despite being reliable sources of information, HCPs failed to make their high-quality content more appealing. This posed a significant challenge to TikTok’s current role as a potential science communication tool. Thus, possibly HCPs could collaborate with NPOs to increase online reach [43]. Audiences should also be more inclined to select videos created by authoritative professionals, rather than opting for more popular content, as studies indicated that video quality and popularity were not necessarily correlated [18, 39, 44].

### Video duration, video quality and popularity

The median duration of TikTok videos related to myopia was 62 s, which was close to the recommended short video length of 60 s [10]. When the video duration exceeded 60 s, a correlation with higher video quality was observed. This can be attributed to the ability of video publishers to present more comprehensive content with longer durations. However, viewer numbers attrition occurred with increasing video length, and longer videos resulted in a smaller proportion of the total video length being watched [42]. This explained the observed decline in audience engagement for videos surpassing 60 s. Wang et al. [39] identified a positive correlation between the video popularity index and the duration of thyroid cancer videos. This association may be influenced by the shorter duration of thyroid cancer videos (37 s). The conclusion drawn from their study may not be directly applicable to myopia videos exceeding 60 s. Acknowledging the contradictory impact of duration on quality and popularity, it is crucial for myopia educators to meticulously prepare condensed and shorter content and minimize the dissemination of ineffective information.

### Video contents, video quality and popularity

Previous studies showed comprehensiveness of video content positively influenced video popularity [40]. Videos received higher reach and engagement when discussing disease prevention, severity, and cues to action [45]. Consistent with these findings, our study revealed a positive correlation between video content addressing myopia risk factors/ -management/outcomes and video popularity, indicated by increased likes and shares. The emotional impact of risk factors and severe outcomes may prompt the audience to seek further information on managing myopia. To improve video reach and popularity, myopia educators should not underestimate the importance of myopia content that readily resonates with audiences, especially when it comes to myopia management content that positively correlated with video quality. In response, video producers should pay more attention to myopia evaluation content that educators frequently overlooked [12].

### Strengths and limitations

Before analyzing the factors related to the quality and audience engagement of myopia-related TikTok videos, we were the first to compare the impact of two search strategies (DCS and MLS) and four sample cut-offs. This comparison mitigated potential selection bias in subsequent analyses. Additionally, the dependent variables included not only video likes and shares but also the rate of likes increment (likes/days), offering a more comprehensive measure of audience engagement. However, limitations also existed. Firstly, various language versions of TikTok have possible discrepancies in their ability to meet the information needs of the public [46]. However, our evaluation was exclusively focused on Chinese myopia videos, which may undermine the generalizability of our findings. Another limitation was related to the DISCERN instrument, originally designed for assessing quality of website information. Although commonly used in TikTok information evaluation [38–40], certain items, such as the inclusion of evidence-based references, were rarely fulfilled in short videos. Additionally, we were unable to access information of video views, posing a limitation in evaluating the popularity of the videos.

## Conclusions

The choice of video retrieval strategy and sample size were found to influence audience engagement but not perceived video quality. Notably, our analysis revealed that HCPs and NPOs generated high-quality videos but with comparatively lower popularity. To enhance the potential for videos to go viral, myopia educators should meticulously produce concise and shorter content (< 60 s) and ensure the sustained production of high-quality videos. Additionally, it is unwise to overlook video content addressing myopia’s risk factors, management, and possible outcomes that readily resonate with audiences, especially myopia management content positively correlated with video quality. For viewers, prioritizing educational myopia information over the pursuit of video popularity and entertaining effects, such as background music, emojis, animation/flash, and placing more emphasis on HCPs and NPOs’ videos and their content might lead to a more efficient acquisition of myopia information.

### Supplementary Information

**Supplementary Material 1.**

**Supplementary Material 2.**

## Data Availability

The data sets generated during and/or analyzed during this study are available from the corresponding author on reasonable request.

## Abbreviations

MLS: “Most-liked” search
DCS: Default comprehensive Search
HCPs: Healthcare professionals
ISCs: Individual science communicators
FPOs: For-profit organizations
NPOs: Non-profit organizations
