# Supplementary material for: Myopia information on TikTok: analysis factors that impact video quality and audience engagement
Source: BMC Public Health. 2024 Apr 29;24:1194. doi: 10.1186/s12889-024-18687-4 (PMC11057166; doi:10.1186/s12889-024-18687-4)
Supplement: Supplementary file 1 — Supplementary Material 1. [file 12889_2024_18687_MOESM1_ESM.docx]

| **Supplemental table S1.** Pairwise comparisons of video engagement variables according to different samples. | | | |
| --- | --- | --- | --- |
| **Pairwise comparisons of video engagement** | | **Adjusted** P (DCS) | **Adjusted** P (MLS) |
| Video likes |  |  |  |
|  | Sample 200 vs 150 | 0.127 | 0.008 |
|  | Sample 200 vs 100 | < 0.001 | < 0.001 |
|  | Sample 200 vs 50 | < 0.001 | < 0.001 |
|  | Sample 150 vs 100 | 0.195 | 0.009 |
|  | Sample 150 vs 50 | 0.004 | < 0.001 |
|  | Sample 100 vs 50 | 0.651 | 0.003 |
| Video comments |  |  |  |
|  | Sample 200 vs 150 | 0.228 | 0.13 |
|  | Sample 200 vs 100 | 0.001 | < 0.001 |
|  | Sample 200 vs 50 | 0.001 | < 0.001 |
|  | Sample 150 vs 100 | 0.388 | 0.118 |
|  | Sample 150 vs 50 | 0.141 | < 0.001 |
|  | Sample 100 vs 50 | 0.999 | 0.174 |
| Video shares |  |  |  |
|  | Sample 200 vs 150 | 0.186 | 0.263 |
|  | Sample 200 vs 100 | < 0.001 | < 0.001 |
|  | Sample 200 vs 50 | < 0.001 | < 0.001 |
|  | Sample 150 vs 100 | 0.278 | 0.255 |
|  | Sample 150 vs 50 | 0.012 | < 0.001 |
|  | Sample 100 vs 50 | 0.927 | 0.052 |
| Video saves |  |  |  |
|  | Sample 200 vs 150 | 0.161 | 0.191 |
|  | Sample 200 vs 100 | < 0.001 | 0.001 |
|  | Sample 200 vs 50 | < 0.001 | < 0.001 |
|  | Sample 150 vs 100 | 0.303 | 0.398 |
|  | Sample 150 vs 50 | 0.006 | < 0.001 |
|  | Sample 100 vs 50 | 0.677 | 0.026 |
