# Supplementary material for: Myopia information on TikTok: analysis factors that impact video quality and audience engagement
Source: BMC Public Health. 2024 Apr 29;24:1194. doi: 10.1186/s12889-024-18687-4 (PMC11057166; doi:10.1186/s12889-024-18687-4)
Supplement: Supplementary file 2 — Supplementary Material 2. [file 12889_2024_18687_MOESM2_ESM.docx]

| **Variables** | | **Likes** | **Likes/days** | **Shares** |
| --- | --- | --- | --- | --- |
|  |  | **β (95% CI)** | **β (95% CI)** | **β (95% CI)** |
| **Video property (Yes = 1, No = 0)** | | | | |
|  | People presence | **3.785 (1.605 to 8.935)** | **4.011 (1.742 to 9.226)** | 1.818 (0.783 to 4.225) |
|  | Marked education | 0.82 (0.429 to 1.568) | 0.676 (0.36 to 1.267) | 0.674 (0.357 to 1.274) |
|  | Background Music | 0.874 (0.451 to 1.694) | 0.787 (0.414 to 1.495) | 1.099 (0.573 to 2.104) |
|  | Emoji | 1.315 (0.65 to 2.664) | 1.405 (0.708 to 2.787) | 0.837 (0.421 to 1.665) |
|  | Animation/flash | 0.78 (0.364 to 1.67) | 0.767 (0.366 to 1.608) | 0.553 (0.263 to 1.164) |
| **Video source** | |  |  |  |
|  | ISCs & FPOs (Ref.) | 0 | 0 | 0 |
|  | HCPs^*^ | 0.156 (0.069 to 0.351) | **0.241 (0.11 to 0.528)** | **0.205 (0.093 to 0.453)** |
|  | NPOs^*^ | 0.433 (0.194 to 0.965) | **0.405 (0.186 to 0.882)** | 0.651 (0.295 to 1.438) |
| **Video duration** | |  |  |  |
|  | < 30 seconds (Ref.) | 0 | 0 | 0 |
|  | 30 - 59 seconds | 1.029 (0.419 to 2.529) | 0.654 (0.273 to 1.564) | 0.829 (0.345 to 1.992) |
|  | 60 - 119 seconds | 0.567 (0.227 to 1.418) | **0.378 (0.155 to 0.919)** | 0.691 (0.282 to 1.694) |
|  | ≥ 120 seconds | **0.171 (0.053 to 0.55)** | **0.166 (0.053 to 0.516)** | 0.334 (0.104 to 1.075) |
| **Video content (2 = Fully mentioned, 1 = Partially mentioned, and 0 = Not mentioned)** | | | | |
|  | Definition | 1.244 (0.766 to 2.018) | 0.977 (0.61 to 1.564) | 1.018 (0.634 to 1.637) |
|  | Signs | 1.543 (1 to 2.382) | 1.404 (0.921 to 2.138) | 1.156 (0.758 to 1.763) |
|  | Risk Factors | **1.606 (1.134 to 2.275)** | 1.358 (0.969 to 1.904) | **1.489 (1.06 to 2.092)** |
|  | Evaluation | 1.02 (0.688 to 1.514) | 1.168 (0.796 to 1.713) | 0.859 (0.583 to 1.265) |
|  | Management | **1.972 (1.309 to 2.971)** | 1.467 (0.985 to 2.186) | **1.642 (1.091 to 2.472)** |
|  | Outcomes | **2.147 (1.419 to 3.248)** | **2.261 (1.513 to 3.384)** | **2.168 (1.445 to 3.258)** |

**Supplemental table S2. the summary statistics (β) of the untransformed data for each audience engagement variable.**

NoteHCPs: healthcare professionals, NPOs: non-profit organizations. ISCs & FPOs: individual science communicators and for-profit organizations. Ref: reference.
